# Defining HIV and SIV Reservoirs in Lymphoid Tissues

**DOI:** 10.20411/pai.v1i1.100

**Published:** 2016-06-14

**Authors:** Claire Deleage, Stephen W. Wietgrefe, Gregory Del Prete, David R. Morcock, Xing Pei Hao, Michael Piatak, Julian Bess, Jodi L. Anderson, Katherine E. Perkey, Cavan Reilly, Joseph M. McCune, Ashley T. Haase, Jeffrey D. Lifson, Timothy W. Schacker, Jacob D. Estes

**Affiliations:** 1 AIDS and Cancer Virus Program, Leidos Biomedical Research, Inc., Frederick National Laboratory for Cancer Research, Frederick, Maryland; 2 Department of Microbiology and Immunology, Medical School, University of Minnesota, Minneapolis, Minnesota; 3 Pathology and Histotechnology Laboratory, Leidos Biomedical Research, Inc., Frederick National Laboratory for Cancer Research, Frederick, Maryland; 4 School of Public Health, Division of Biostatistics, University of Minnesota, Minneapolis, Minnesota; 5 Division of Experimental Medicine, Department of Medicine, University of California, San Francisco, California; 6 Department of Medicine. Medical School, University of Minnesota, Minneapolis, Minnesota; 7 Deceased 19 September 2014

**Keywords:** HIV, SIV, reservoir, follicular dendritic cells, B cell follicles, *in situ* hybridization, RNAscope, DNAscope

## Abstract

A primary obstacle to an HIV-1 cure is long-lived viral reservoirs, which must be eliminated or greatly reduced. Cure strategies have largely focused on monitoring changes in T cell reservoirs in peripheral blood (PB), even though the lymphoid tissues (LT) are primary sites for viral persistence. To track and discriminate viral reservoirs within tissue compartments we developed a specific and sensitive next-generation *in situ* hybridization approach to detect vRNA, including vRNA+ cells and viral particles (“RNAscope”), vDNA+ cells (“DNAscope”) and combined vRNA and vDNA with immunohistochemistry to detect and phenotype active and latently infected cells in the same tissue section. RNAscope is highly sensitive with greater speed of analysis compared to traditional *in situ* hybridization. The highly sensitive and specific DNAscope detected SIV/HIV vDNA+ cells, including duplexed detection of vDNA and vRNA or immunophenotypic markers in the same section. Analysis of LT samples from macaques prior to and during combination antiretroviral therapy demonstrated that B cell follicles are an important anatomical compartment for both latent and active viral persistence during treatment. These new tools should allow new insights into viral reservoir biology and evaluation of cure strategies.

## INTRODUCTION

Due to the accessibility and ease of sampling peripheral blood (PB) in a clinical research setting and the prevailing thought that PB accurately mirrors systemic human immunodeficiency virus (HIV)/simian immunodeficiency virus (SIV) infection and dynamics, most studies of the size, decay kinetics, and features of viral reservoirs in HIV-infected individuals have relied on the longitudinal monitoring of plasma viral loads and infected cell subsets within PB mononuclear cells (PBMCs), including latently infected resting CD4+ T cell subsets [1]. However, since HIV/SIV infections are primarily diseases of lymphoid tissues (e.g., lymph nodes, spleen, mucosal-associated lymphoid tissue [MALT]) in which the vast majority of HIV/SIV-infected cells and viral repositories reside [2–11], the assumption that the PB accurately reflects what goes on within these tissues is largely conjecture. We therefore continue to evaluate the lymphatic organ system itself largely by *in situ* technologies [9] as an essential component of a comprehensive assessment of viral reservoirs and persistence *in vivo* and to fully recognize the therapeutic potential of HIV-1 curative strategies.

The assessment of HIV/SIV viral reservoir size and phenotype has primarily been performed by approaches that require disruption of the tissue and/or preparation of single cell suspensions so that quantitative measurements can be performed [12, 13]. In addition to losing important spatial information, the processing of cells from whole tissues may result in: i) misinterpretation of cell phenotypes (i.e. cell surface marker expression), ii) changes in viral expression patterns, iii) limited recovery of certain tissue resident cells, and iv) loss of cells due to processing induced death. Thus, while these approaches will continue to provide valuable information, we and others [14] strive to develop and use novel *in situ* technologies to visualize and quantify HIV-1 and SIV infections in anatomically intact native tissue environments to understand the types of cells and anatomic structures in which the virus is produced and how it is stored in follicles and persists in latently or covertly infected cells [3, 5, 8–11, 15–18].

While these classic *in situ* technologies remain to be valuable in characterizing SIV and HIV-1 infection and persistence in lymphoid tissues (LT), there is ample room for improvement in approaches that are less labor intensive, simpler, and faster than current *in situ* hybridization (ISH) methods with radiolabeled probes or chromogenic detection; more facile and reproducible than *in situ* Polymerase chain reaction (PCR) approaches in routinely detecting vDNA+ cells in formalin-fixed paraffin embedded (FFPE) tissues, a prerequisite for detection of latently infected cells *in situ* [3, 14]; and approaches to simultaneously detect vRNA and vDNA in the same tissue section as a valuable tool to identify covertly infected transcriptionally inactive vDNA+ / vRNA-cells in tissues. We show here: 1) that an optimized next-generation ISH platform (termed RNA-scope [19, 20]) for the rapid detection of vRNA (with results obtained within 1 day) has sufficient sensitivity to reliably detect single virions in B cell follicles (BCF) in FFPE tissue sections, 2) that an approach for the detection of vDNA *in situ* (referred to as DNAscope) reliably and readily detects vDNA+ cells, and 3) that we have developed an *in situ* method to simultaneously visualize vRNA and vDNA in the same tissue section and thereby identify transcriptionally latent infections (vDNA+/vRNA-cells) in LTs. These new, highly sensitive *in situ* hybridization approaches applied to LT samples from macaques prior to and during combination antiretroviral therapy (cART) document the importance of BCFs in active, latent, and persistent infections during treatment. These data underscore the utility of new and sensitive ISH tools that can provide additional insight into viral persistence, reservoir establishment, tissue compartmentalization, and reservoir phenotype in the local *in vivo* tissue environment.

## RESULTS

### Sensitive detection of SIV vRNA+ cells and viral particles by next-generation RNAscope ISH

**Figure 1.**
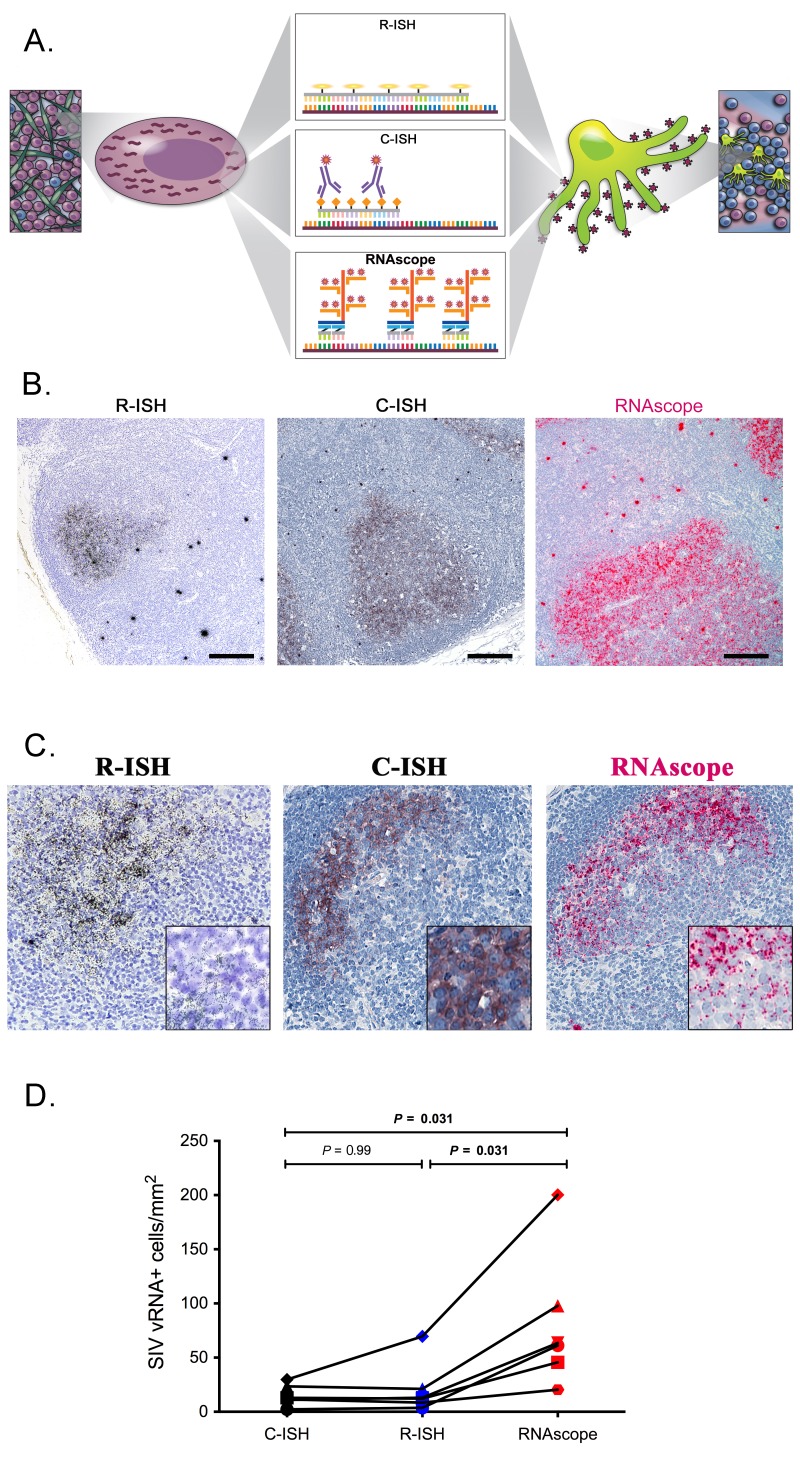
**Increased sensitivity to detect vRNA by the next-generation RNAscope approach compared to standard in situ hybridization (ISH) approaches.** (A) Schematic to demonstrate key differences between standard radiolabeled ISH (R-ISH), chromogenic ISH (C-ISH) and the next-generation RNA-scope approaches, with cartoon representation of a productively infected vRNA+ cell on the left and virion trapped on FDCs on the right. Comparison between the gold-standard R-ISH, C-ISH, and RNAscope ISH techniques at low (B) and high magnification (C) demonstrating the exquisite sensitivity with potential for detection of virions in tissues. Scale bars = 200 μm. (D) Quantitative image analysis of subjacent lymph node sections from 6 chronically infected SIV+ RMs demonstrating a trend for increased sensitivity of RNAscope for detecting vRNA+ cells. *P* values were based on the Wilcoxon-sign rank test corrected for multiple comparisons.

ISH methods offer the potential to provide key information about the status of both residual latent infection and active viral replication in the setting of cART, including anatomic information not readily obtained by other methods of virological analysis. To better understand, track and discriminate viral reservoirs within tissue compartments, we first compared the sensitivity and specificity of two current ISH approaches (radiolabeled [R-ISH]—the current “gold standard”—and chromogenic [C-ISH]) to a new next-generation technology (RNAscope), evaluating their utility for detecting vRNA+ cells and vRNA in virions in the follicular dendritic cell network (FDCn) in tissues from SIV-infected rhesus macaques (RMs) (Figure 1A). Productively infected vRNA+ cells typically have a densely staining spherical signal that encompasses the entire cell body, while virions trapped on the FDCn typically have a diffuse lattice-like signal pattern within B cell follicles (BCF) (Figure 1B, C) [5]. Quantitative image analysis of lymph node sections from 6 SIV-infected RMs at 6-weeks post-infection (wpi) demonstrated no significant difference in the detection of productively infected vRNA+ cells in viremic macaques between R-ISH and C-ISH techniques using standard conditions (Figure 1D). The next-generation RNAscope approach was as sensitive in detecting productively infected vRNA+ cells compared to both R-ISH and C-ISH techniques, with a trend to greater sensitivity that while not statistically significant (*P* = 0.094) (Figure 1D) is consistent with identifying vRNA+ cells with low signal as a result of the more aggressive retrieval process and remarkably low background in the RNAscope protocol. Most important, this next-generation ISH technology was significantly more rapid than either the C-ISH or the gold standard R-ISH approaches, with an assay time of only 8 to 9 hours, compared to 3 days for C-ISH and 6 to 21 days for R-ISH (depending on the length of exposure).

**Figure 2.**
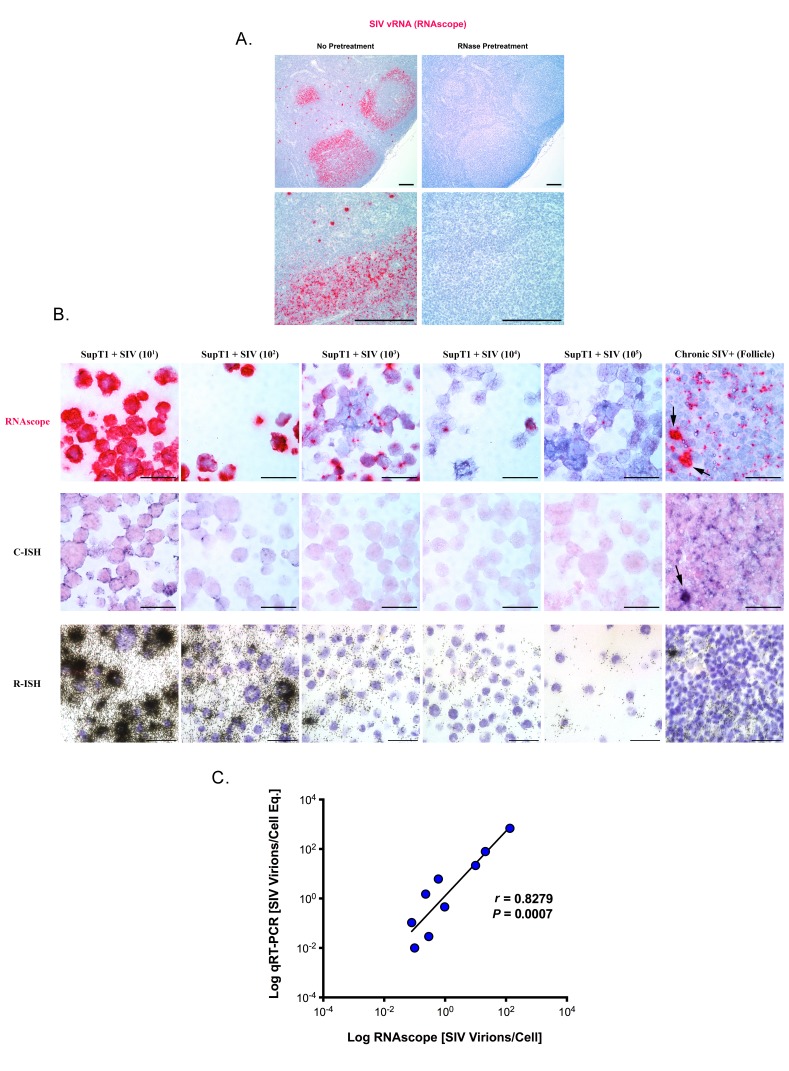
**Next-generation RNAscope ISH is capable of detecting individual viral particles in tissues.** (A) Low (top panel) and high (bottom panel) magnification images of a lymph node from chronically SIV+ rhesus macaque (RM) showing vRNA+ cells and follicular dendritic cells (FDC)-bound viral particles within the B cell follicle (BCF) (left panel) and the complete loss of signal after RNase pretreatment (right panel). Scale bars = 200 μm. (B) SIV binding to the surface of SupT1 CCR5-Hi cells. Images from 10-fold dilutions of concentrated SIV demonstrating increased detection of individual viral particles by RNAscope (top panel) compared to less discriminating chromogenic *in situ* hybridization (C-ISH) (middle panel) and radiolabeled *in situ* hybridization (R-ISH) (bottom panel). Right panel shows high magnification images of chronic SIV+ RM lymph node from subjacent sections showing productively infected vRNA+ cells (arrows) and individual viral particles by RNAscope (top), but lack of clear viral particle discrimination using C-ISH (middle) and R-ISH (bottom). Scale bar = 50 μm. (C) The number of viral particles bound to SupT1 CCR5-Hi cells quantified by qRT-PCR is strongly correlated to the number of virions quantified by RNAscope. Data were log10 transformed, and *P* values were based on associations between paired comparisons using the Pearson's Correlation test.

**Supplemental Figure 1.**
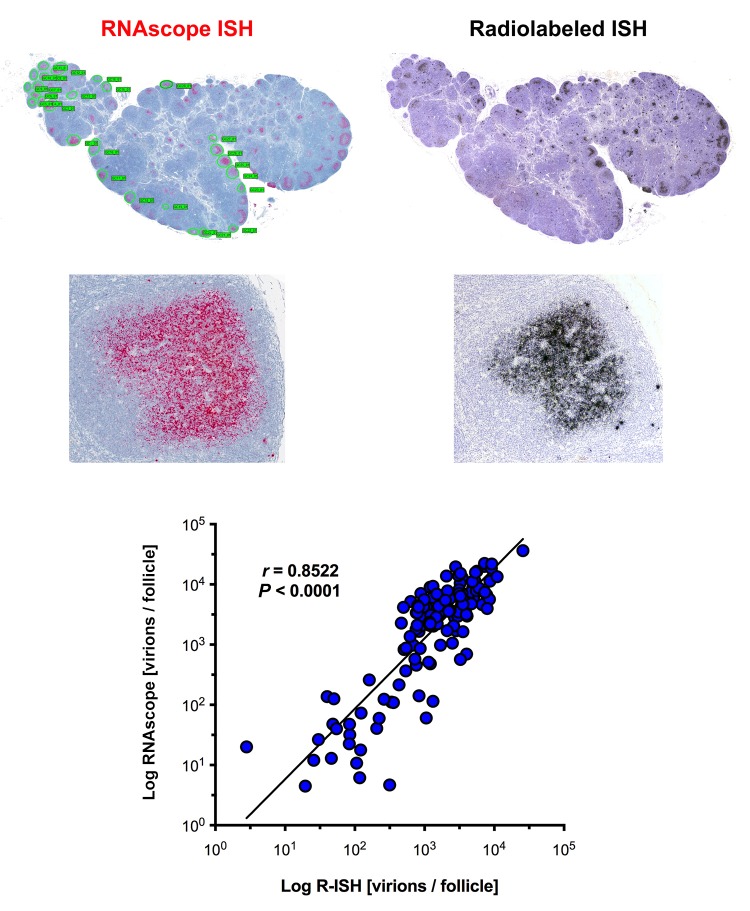
**Comparison of RNAscope and R-ISH approaches for detection of viral particles in B cell follicles.** Representative subjacent whole lymph nodes (LN) sections (top panel) and high magnification (lower panel) images showing SIV vRNA expression following RNAscope (left panel) and radiolabeled (right panel) *in situ* hybridization (ISH). Viral particles were quantified from 163 mapped B cell follicles in 3 subjacent lymph node tissue sections from 3 chronically SIV+ RMs. There is a strong correlation between the enumerated virions quantified by R-ISH and RNAscope ISH in B cell follicles. Quantified values were log10 transformed and *P* values were based on associations between paired comparisons using the Pearson's Correlation test.

The RNAscope ISH approach, like R-ISH and C-ISH, also detected vRNA in virions on the FDCn within the BCF of chronically SIV-infected RMs; however, RNAscope provided superior visual discrimination of putative viral particles compared to other ISH approaches (Figure 1C). Pre-treatment of tissue sections with RNase completely ablated vRNA detection of both productively infected cells and punctate signal from putative virions within BCF (Figure 2A). The relationship between the estimated number of virions within subjacent follicles of chronic SIV+ RMs derived by R-ISH and RNAscope approaches was linear and highly correlated (Supplemental Figure 1), demonstrating RNAscope as a facile and highly sensitive ISH approach for the detection of vRNA within the FDCn *in vivo*.

**Supplemental Figure 2.**
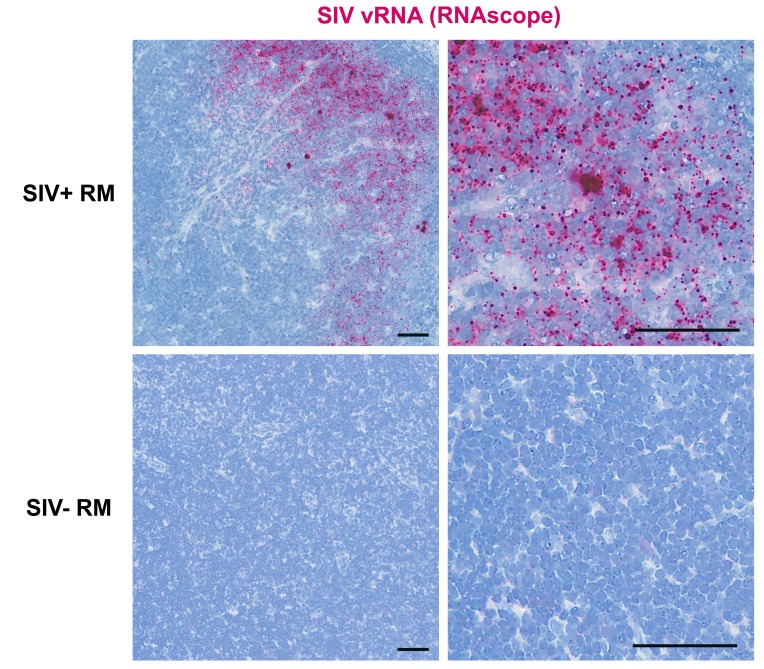
**Specificity of RNAscope for the detection of vRNA+ cells and viral particles.** Representative low and high magnification RNAscope *in situ* hybridization (ISH) images from a chronically SIV-infected rhesus macaque (RM) (top panel) and a SIV-negative RM (bottom panel) demonstrating the robust detection of SIV vRNA only SIV-infected, but not in SIV-negative lymphoid tissues. Scale bars = 50 μm.

For quantitative validation of the RNAScope method in detecting viral particles, we documented detection of virions in a binding experiment in which SupT1 CCR5-Hi cells were incubated with serial dilutions of concentrated highly purified infectious SIV (sucrose double banded virus), followed by parallel analysis using both qRT-PCR and RNAscope ISH with quantitative image analysis, as well as by comparative analysis with R-ISH and C-ISH. Bound virions were visualized as an intense red signal on SupT1 CCR5-Hi cells with high concentrations of virus after RNAscope ISH, which decreased to a discrete individual punctate signal in proportion to the dilution of SIV added to SupT1 CCR5-Hi cells (Figure 2B). While the gold standard R-ISH was similar to RNA-scope in detecting virus bound to SupT1 CCR5-Hi cells down to a dilution of 10^5^, the RNAscope method provided superior visual discrimination of viral particles bound to these cells, visualized as a single punctate signal compared to the diffuse silver grain signal surrounding cells using the R-ISH method. Both RNAscope and R-ISH were about 3-orders of magnitude more sensitive at detecting viral particles bound to SupT1 CCR5-Hi cells than the C-ISH method. The estimated number of virions bound per cell, as determined by RNAscope analysis, was highly correlated with vRNA levels determined by qRT-PCR results over several orders of magnitude (Figure 2C). The robustness and specificity of detecting vRNA+ cells and viral particles by RNAscope ISH was determined by quantifying the relative false detection rate in lymphoid tissues from SIV-negative RMs (Supplemental Table 1; Supplemental Figure 2). We analyzed 274 high power regions of interest (ROIs) consisting of nearly 70 mm^2^ of tissue and found only 2 false positive “vRNA+ cells” and 4 false positive “virions.”

### Utility of the next-generation RNAscope ISH in a cART SIV NHP model

**Figure 3.**
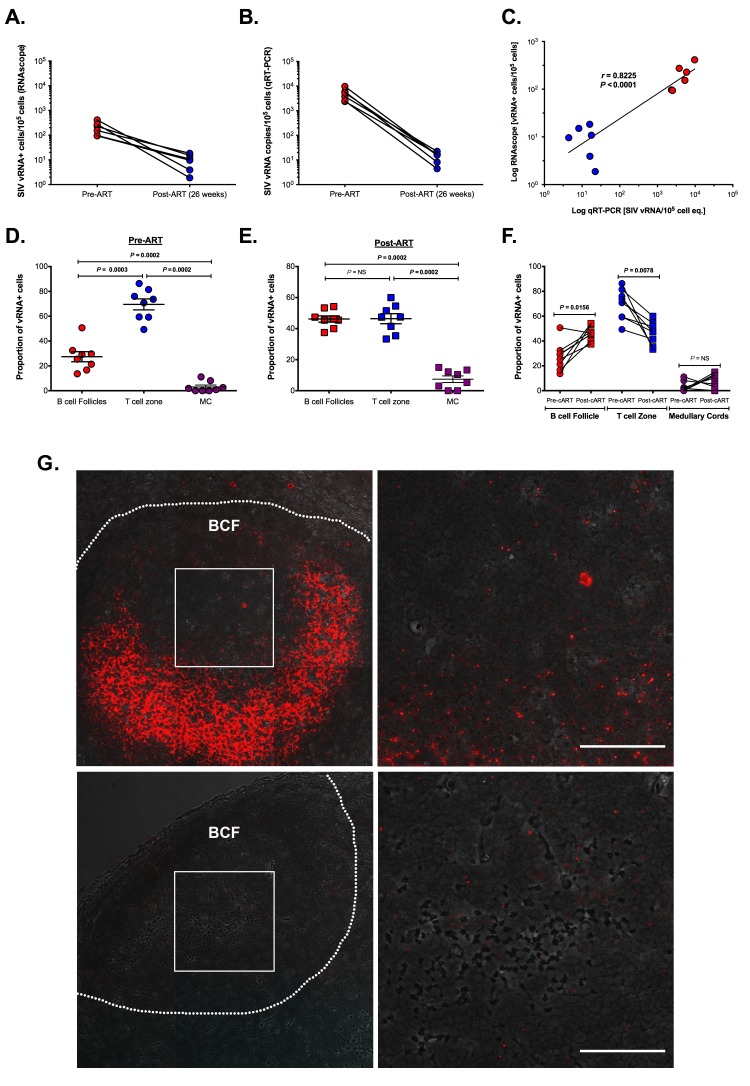
**Utility of RNAscope for the detection and quantification of vRNA+ cells in a combination antiretroviral therapy (cART) SIV NHP model with strong correlation to qRT-PCR.** (A) The number of vRNA+ cells quantified by RNAscope and (B) the number of vRNA copies quantified by qRT-PCR are (C) strongly correlated before and during cART. Data were log10 transformed and *P* values were based on associations between paired comparisons using the Pearson's Correlation test. (D) The proportions of vRNA+ cells within distinct secondary lymphoid tissue anatomical sites (i.e., B cell follicles (BCF), T cell zone, and medullary cords) before and (E) during cART. *P* values were based on the Mann-Whitney test. (F) The frequency of vRNA+ cells in BCF increased, while that of vRNA+ cells in T cell zone decreased. The proportion of vRNA+ cells within the MC is unchanged, suggesting a steady rate of cell trafficking. *P* values were based on the Wilcoxon matched-pairs signed rank test. (G) RNAscope in situ hybridization (ISH) has the ability to detect low-level vRNA+ cells (not shown) and FDC-bound viral particles in chronically SIV+ RMs before (top panel) and after 26 weeks of cART (bottom panel). Scale bars = 50 μm.

**Supplemental Figure 3.**
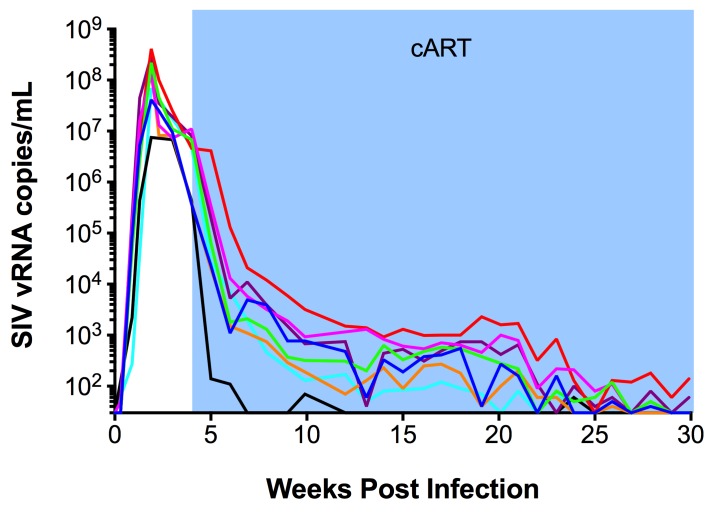
**Plasma viral load measures from SIV-infected RMs.** Eight rhesus macaques (RMs) were infected with SIVmac239 and placed on combination antiretroviral therapy (cART) after 4 weeks post infection (wpi) and followed for an additional 26 weeks (30 wpi).

Having validated the sensitivity and specificity of next-generation RNAscope ISH, we applied this approach to tracking the virological response to cART in lymph node samples obtained from SIV+ RMs before and after 26 weeks of cART and compared these results to parallel measurements performed by qRT-PCR. Eight RMs were infected with SIVmac239 and started on cART at 4 wpi; lymph node biopsies were taken prior to cART initiation (4 wpi) and after 26 weeks of cART (30 wpi), at which time 6 of the 8 RMs had plasma viral loads below assay limits (< 30 SIV vRNA copies/ml) (Supplemental Figure 3). The number of productively SIV vRNA+ cells/10^5^ cells measured by RNAscope ISH and vRNA copies/10^5^ cell equivalents measured by qRT-PCR from the same lymph node biopsy (RNA extracted from bulk tissue) decayed in parallel and were highly correlated (Figure 3A-C). RNAscope ISH provided additional anatomical information on the location of SIV vRNA+ cells and the proportional change associated with cART (Figure 3D-F). Prior to cART (4 wpi), on average 69% of the SIV vRNA+ cells were located in the paracortical T cell zone (TCZ) with an average 27% located within BCF and only an average 3% were located within the medullary cords (MC) of the lymph nodes. The differences between the frequencies of vRNA+ cells within each anatomical site were highly statistically significant before cART (Figure 3D-F). After cART the total number of vRNA+ cells decreased dramatically, with the relative proportion of the remaining vRNA+ cells decreased in the TCZ and the proportion increased in the BCF until there was no significant difference in the proportion of SIV vRNA+ cells within these two areas, with an average 46% of SIV vRNA+ cells located in each distinct anatomical environment (Figure 3E-F). Although this change in the proportion of SIV vRNA+ cells within the TCZ and BCF after cART was statistically significant; there was no significant change in the proportion of vRNA+ cells within the MC (Figure 3F). These results are consistent with persistent low-level viral expression during cART as has previously been reported in HIV-infected cART-suppressed patients [15] and highlight the BCF as a particularly important anatomic site for infected cells during treatment. Consistent with the importance of BCF in viral persistence, we also found that virions associated with the FDCn remained detectable after 26 weeks of cART, albeit at very low levels compared to pre-cART time points (Figure 3G). While the decrease in virions measured after 26 weeks of cART within BCFs was on average 99.5%, nearly every follicle analyzed had infrequent but discernable virions within BCFs by RNAscope ISH (Figure 3G), which is again consistent with residual and potentially infectious virus in the FDCn pool at this time as has been shown recently for HIV in well-suppressed patients on cART with undetectable virus in PB [21].

### Detection of vDNA in situ using next-generation DNAscope ISH

**Figure 4.**
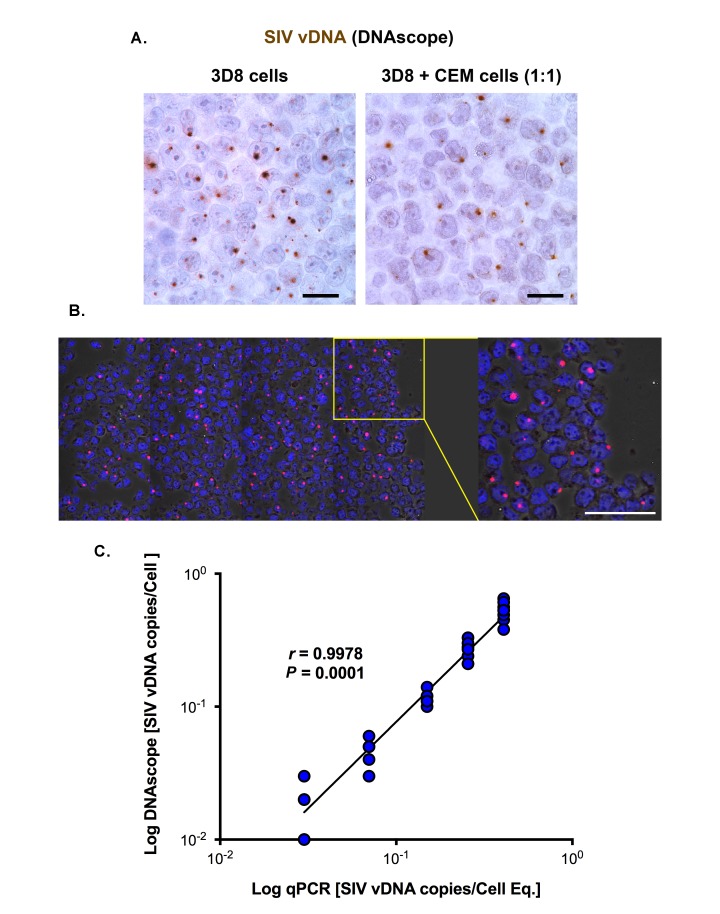
**Viral DNA detection in 3D8 cells.** (A) Brightfield (scale bar = 20 μm) and (B) confocal (scale bar = 50 μm) 3D8 cell analysis demonstrating vDNA detection specifically within the nucleus of cells with primarily 1 integrated copy of SIV. (C) There is a strong correlation between the quantification of 3D8 cells diluted into CEM cells determined by DNAscope and qPCR. Each symbol represents an individual high magnification image quantified from a single experiment. One of three experiments shown. Data were log10 transformed and *P* values were based on associations between paired comparisons using the Pearson's Correlation test.

**Supplemental Figure 4.**
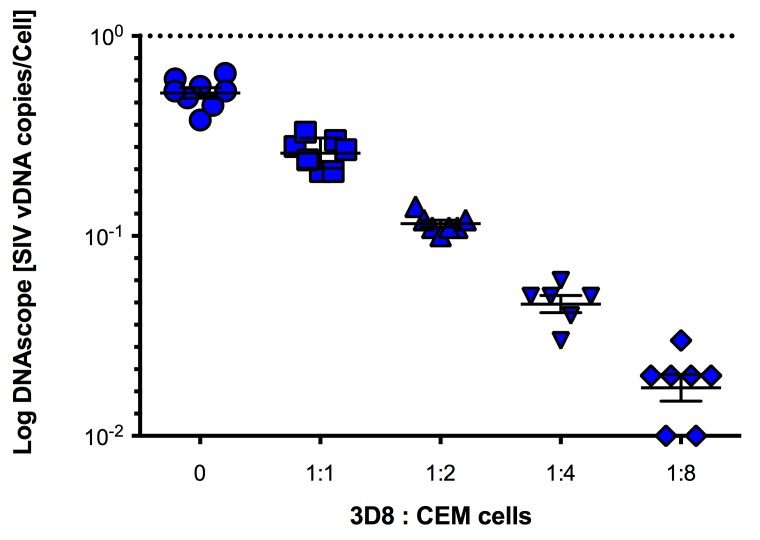
**DNAscope quantification of vDNA in 3D8 cells diluted in uninfected CEM cells.** DNAscope was performed on 3D8 cells diluted into CEM cells. Each symbol represents an individual high magnification image quantified from a single experiment. One of three experiments shown.

**Supplemental Figure 5.**
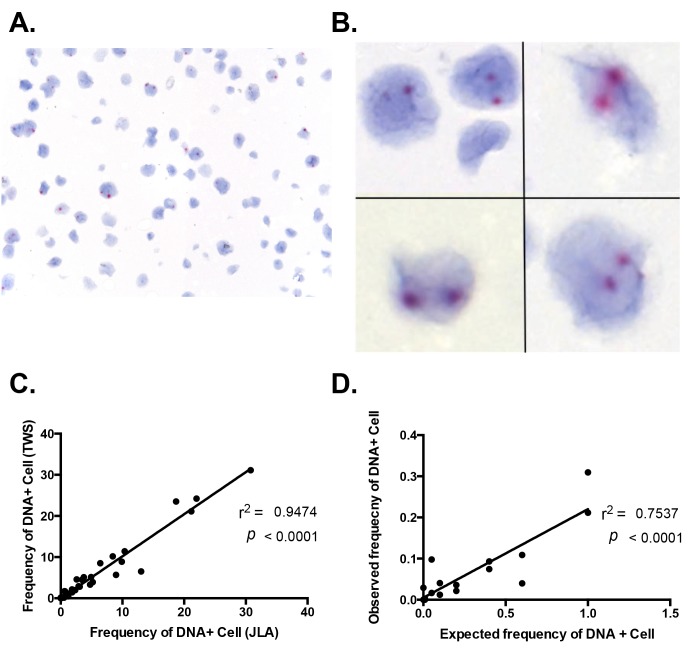
**Detection and quantification of HIV-1 vDNA in ACH-2 cells.** Low (A) and high (B) magnification images showing HIV-1 vDNA (Red) by DNAscope in ACH-2 cells. Note that the nuclei of many ACH-2 cells contain multiple copies of vDNA, consistent with multiple integration sites per ACH-2 cell. (C) Quantification is highly reproducible between independent blinded users. (D) Strong correlation between the actual and expected frequencies of vDNA+ cells.

The detection of individual virions by the RNAscope ISH approach encouraged us to explore the potential to detect vDNA+ cells in fixed tissues with this technology, a task beyond the capability of current conventional ISH methods. To test this, we used the 3D8 cell line, derived from chronically-SIVmac316 infected CEM×174 cells with reported 1 copy of integrated SIV DNA[22] and the ACH-2 cell line, a latently infected T cell clone initially reported to contain 1 integrated proviral copy of HIV-1 per cell, derived from A3.01 cells that were infected with HIV-LAV and cloned by limiting dilution [23–25]. We modified the RNAscope protocol using sense probes to detect SIV and HIV-1 vDNA in these cell lines by “DNAscope” (see Methods) and analyzed serial dilutions of 3D8 and ACH-2 cells in uninfected CEM cells. DNAscope ISH detected vDNA localized within the nuclei of 3D8 cells by both bright field and fluorescence confocal analysis (Figure 4A-B). The frequency of cells scored as vDNA+ decreased as the proportion of serially diluted 3D8 infected cells present in a constant number of total cells (infected plus uninfected CEM cells) decreased (Supplemental Figure 4). In addition, the number of vDNA+ cells detected by DNA-scope ISH was strongly correlated with the number of vDNA copies per cell equivalent detected by parallel qPCR analysis. (Figure 4C, D). Similar data were obtained with ACH-2 cells and HIV-1 DNAscope ISH (Supplemental Figure 5). Interestingly, while we usually found one punc-tate signal of vDNA within 3D8 cells, ACH-2 cells frequently contained more than one distinct vDNA signal in the nucleus by DNAscope suggesting that ACH-2 cells frequently contain multiple integration sites as previous reported [26] (Supplemental Figure 5). Assuming the average diameter of CEM cells to be 15 μm [27], and DNAscope measurements having been performed on 4 to 6 μm thick sections, we would expect to detect a vDNA signal in 21% to 29% of undiluted 3D8/ACH-2 cells, in agreement with measurements for both 3D8 and ACH-2 cells (Supplemental Figures 4–5). We calculated the percent by computing the probability that the nucleus is contained in a cut section conditional on the cell being in the cut section. We calculated the percent by computing the probability that the nucleus is contained in a cut section conditional on the cell being in the cut section (i.e. the ratio of the probability of the 2 events). If we assume a cut cell pellet sample has thickness 2*T* and the cut sections are 2*s* thick, then if we assume that the nuclei of cells are uniformly distributed throughout the section we find that the probability that a cell nucleus is in the cut section is *s*/*T*. The probability that a cell intersects a section is the probability that a cell is within a cell diameter (denoted *d*) of the cut section, hence if we assume that cells are uniformly distributed throughout the cut-cell pellet section we find that this probability is (2*s*+*d*)/(2*T*). Combining these results we get the expression 1/(1+*d*/(2*s*). So if the cell radius is 7.5 and the section thickness is 4 to 6 μm we get the percent range given above. A 95% confidence interval for the regression coefficient we obtain from regressing the counted values on the expected values is (0.15, 0.28), which is consistent with our calculations.

### Utility of the next-generation DNAscope ISH for the detection of vDNA+ cells in a cART SIV NHP model

**Figure 5.**
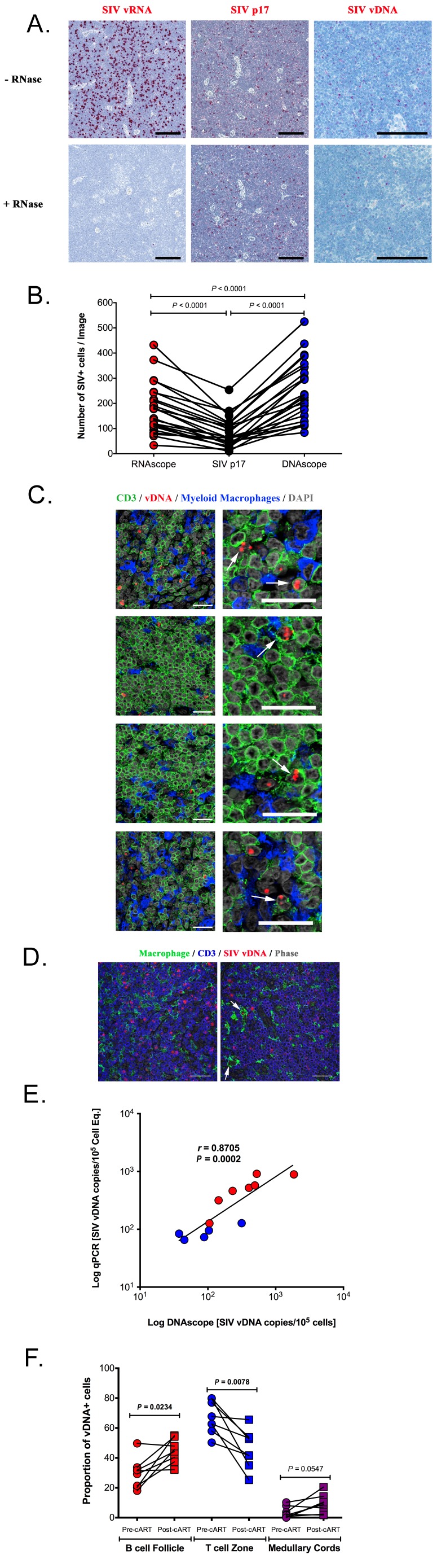
**Characterization of vDNA+ cells in secondary lymphoid tissues *in vivo*.** (A) Visual and (B) quantitative comparison of DNAscope to RNAscope and IHC for SIVp17 protein using an SIV-infected (acute) NHP model showed no effect of RNase treatment in the ability to detect vDNA *in situ,* whereas RNAscope detection of vRNA is highly sensitive to RNase pretreatment. *P* values were based on the Mann-Whitney test. Scale bars = 200 μm. (C) Confocal analysis of an acutely SIV-infected lymph node demonstrating the ability to combine vDNA detection (red) with cell phenotypic immunofluorescence analysis of CD3+ T cells (green), and CD68+/CD163+ myeloid lineage cells (blue) demonstrating the predominant infected cell type to be T cells. Arrows point to examples of “superinfected” T cells that contain multiple vDNA copies per cell. Scale bars = 25 μm. (D) Confocal analysis of an acutely SIV-infected lymph node demonstrating rare vDNA detection (red) in CD68+/CD163+ myeloid lineage cells (green) demonstrating the predominant infected cell type to be T cells (blue). Scale bars = 25 μm. (E) Strong statistically significant correlation of the number vDNA+ cells before and during cART, as determined by DNAscope and qPCR quantification on a per cell basis. Data were log10 transformed and *P* values were based on associations between paired comparisons using the Pearson's Correlation test. (F) Visualization and quantification of vDNA+ cells by DNAscope allowed for the discrimination of the anatomical location of vDNA+ cells within intact tissues. Using an SIV RM model of cART we noted significant changes in the proportions of vDNA+ cells within the distinct lymph node anatomical sites, similar to results seen in vRNA+ cell frequency changes reported in Figure 4. *P* values were based on the Wilcoxon matched-pairs signed rank test.

Having validated the ability of DNAscope to detect vDNA in cell lines, we used the DNAscope ISH approach to detect vDNA in cells in lymph node tissue sections from acutely SIV infected RMs and compared these results to RNAscope detection of vRNA and SIVp17 protein detection by immunohistochemistry (IHC). RNAscope yielded robust detection of vRNA+ cells, virions (data not shown), and IHC, while less sensitive, was capable of detecting abundant SIVp17+ cells in acutely SIV-infected RMs (Figure 5A-B). Furthermore, DNAscope ISH readily detected vDNA in lymph nodes from acutely SIV-infected RMs (Figure 5A-B). Most important, while RNase pre-treatment completely ablated vRNA detection by RNAscope, both IHC detection of SIVp17+ cells and DNAscope ISH detection of vDNA+ cells were unaffected by RNase pre-treatment (Figure 5A), demonstrating that DNAscope ISH specifically detects vDNA in cells in tissue sections.

**Supplemental Figure 6.**
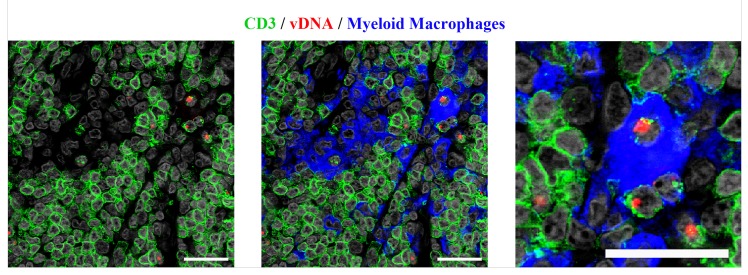
**Macrophage phagocytosis of vDNA+ T cells in secondary lymphoid tissues *in vivo*.** Confocal analysis of an acutely SIV-infected lymph node showing vDNA detection (red) with cell phenotypic immunofluorescence analysis of CD3+ T cells (green) and CD68+/CD163+ myeloid lineage cells (blue), demonstrating (arrows) the detection of macrophages that have ingested vDNA+ T cells (arrows). Scale bars = 25 μm.

**Supplemental Figure 7.**
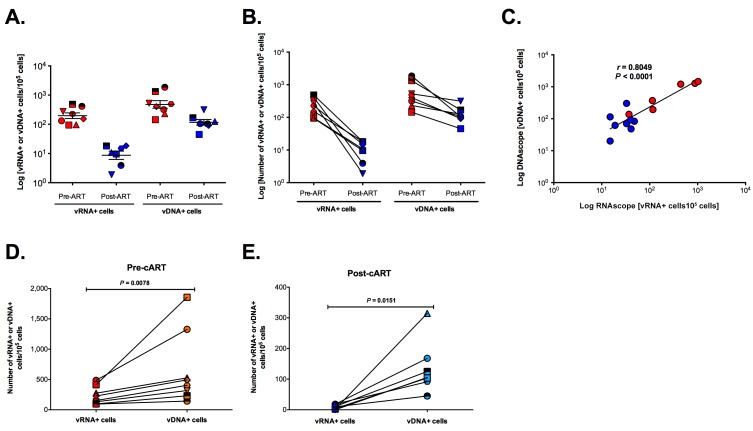
**Effect of cART on the number of vRNA+ and vDNA+ cells in secondary lymphoid tissues *in vivo*.** (A) The mean change in vRNA+ and vDNA+ cells before and during 26 weeks of combination antiretroviral therapy (cART). (B) The individual animal change in vRNA+ and vDNA+ cells before and during 26 weeks of cART. (C) The relationship between vDNA and vRNA before and during cART. (D) The comparison in the number of vRNA+ and (E) vDNA+ cells before and during 26 weeks of cART.

DNAscope can also be combined with immunofluorescence detection for phenotypic analysis of the infected cell type. In acutely SIV-infected RMs the vast majority of vDNA+ cells in lymph nodes were T cells (Figure 5C), and many infected T cells at the time of peak infection contained 2 or more distinct punctate vDNA signals within their nuclei. This finding is consistent with similar results in ACH-2 cells and with several reports of superinfection of cells through virological synapses mediated by cell-to-cell transmission [28, 29] (Figure 5C-D). There were vDNA+ macrophages that appeared to be authentically infected (Figure 5D), but the vDNA detected in other macrophages was associated with ingested infected T cells (Supplemental Figure 6), as has been previously described [30]. We quantified vDNA+ cells before and after cART in 8 RMs, as described in Figure 4, and compared these results to qPCR results from the same tissues. There was a strong correlation between the number of vDNA+ cells quantified by qPCR and DNAscope (Figure 5E), with a decrease in the total number of vDNA+ cells after 26 weeks of cART. In addition, DNAscope revealed a proportional enrichment of the remaining vDNA+ cells within the BCF relative to other lymph node anatomic sites after suppressive cART, similar to the vRNA+ cell results, once again highlighting the BCF as an important site of viral persistence during cART (Figure 5F). Consistent with vDNA+ cells being more stable during long-term cART than vRNA+ cells, we see an average of approximately 5-fold reduction in vDNA+ cells compared to about a 24-fold reduction in vRNA+ cells after 26 weeks of cART, but a strong association still exists between the number of vRNA+ and vDNA+ cells before and during cART (Supplemental Figure 7). We have generated, validated, and applied both the RNAscope and DNAscope approaches for the detection of HIV-1 vRNA and vDNA in clinically relevant patient samples infected with a multitude of HIV-1 clades (data not shown), and these HIV-1 lineage-specific reagents are now available to the scientific community.

### Duplexed RNAscope / DNAscope ISH for the combined detection of vRNA and vDNA

**Figure 6.**
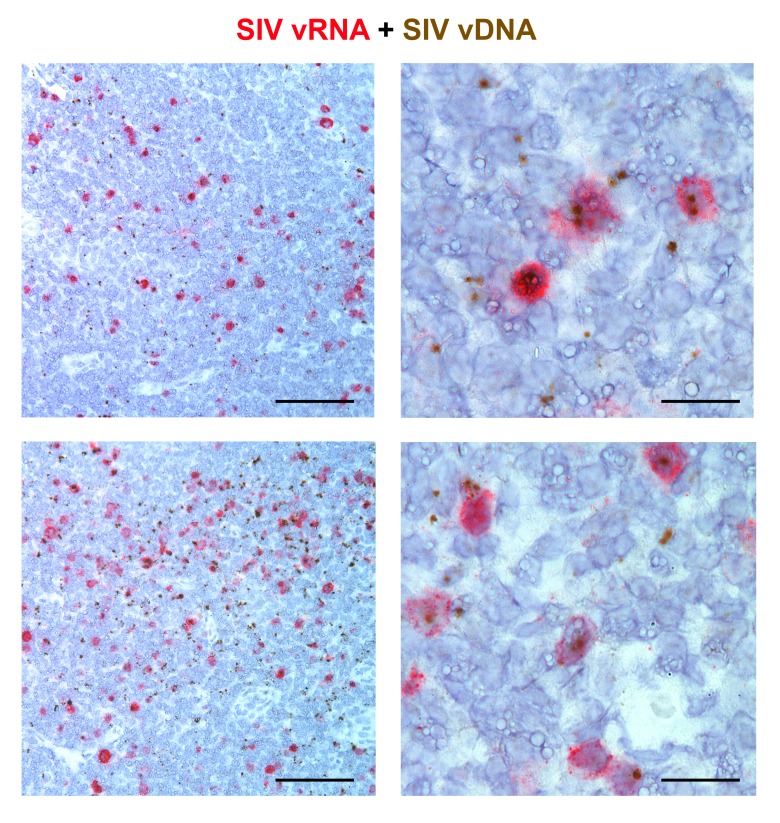
**Duplex Viral RNA and vDNA detection in the same tissue section.** Representative low (left panel; scale bar = 200 μm) and high magnification (right panel; scale bar = 50 μm) images demonstrating the combination of both RNAscope and DNAscope in a chronically SIV-infected RM lymph node demonstrating the ability to detect vRNA and vDNA in the same tissue section and providing a powerful approach to identify transcriptionally silent vDNA+ vRNA-cells *in situ*.

Finally, we reasoned that the ability to unambiguously identify and quantify latently HIV/SIV infected cells that are transcriptionally silent in their native *in vivo* environment could provide needed insight into reservoir persistence and maintenance; thus, we developed an approach that combined RNAscope with DNAscope. This combination approach used sense probes (vDNA) targeting the 5′ gag-pol portion of the SIV/HIV genome and anti-sense probes (vRNA) targeting genes in the 3′ half of the genome (vif, vpx, vpr, tat, env and nef), as well as the TAR element in the 5′ genome (Supplemental Table 3). This approach was capable of distinguishing transcriptionally active cells (vRNA+, vDNA+) from transcriptionally inactive, potentially latent, infected cells or cells harboring transcriptionally incompetent proviruses (vRNA-, vDNA+) in the same tissue section (Figure 6).

## DISCUSSION

The persistence of HIV-1 reservoirs despite apparently effective cART remains a formidable obstacle to achieving sustained virologic remission in HIV-infected individuals in the absence of continued cART [1, 31–33]. While the resting CD4+ T-cell reservoir in PB has been the most intensely studied and characterized HIV-1 reservoir to date, it has long been known [34] that the LT are the principal site [2, 3, 5, 9–11] where virus is produced, that the virus is stored in the FDCn in quantities estimated to be 40- to 50-fold larger than in PB [5], and that it is harbored as transcriptionally inactive proviruses in CD4+ T cells with population sizes of the order of 10^10^ cells in an untreated 70-kg individual [3, 9]. It is thus not surprising given the large number of cells with potentially reactivatable proviruses, continuing replication in LT under conditions of relatively low levels of ARVs [15, 35] and infectious virions in the FDCn pool, that recrudescent infection on treatment interruption is rapid and multifocal in the LT reservoir [16].

It also follows that experimental approaches to tracking this LT reservoir are essential in assessing strategies to achieve sustained remissions when cART is stopped. To that end, we describe here fast and facile *in situ* technologies to track productively infected cells, the FDCn pool and cells that harbor transcriptionally inactive proviruses before and during cART, and the changes effected by a reactivation strategy, e.g., increases in the proportion of vDNA+ and vRNA+ cells. While *in situ* PCR was developed to detect a single copy of lentiviral DNA in a cell [36] and has been used with ISH to reveal the large population of covertly infected vDNA+ vRNA-CD4+ T cells in LT in HIV-1 infected individuals [4], the DNAscope and combined DNAscope/RNAscope methods with the ability to detect vDNA and vRNA in a single tissue section are significant advances in assessing the effectiveness of reactivation strategies *in vivo*. The remarkable specificity of this approach with little-to-no background “noise” is likely due to the requirement of a pair of target double Z probes (ZZ), each possessing a different type of tail sequence, being directly juxtaposed to each other forming a contiguous target region for subsequent hybridization. This specificity coupled with the exceptional sensitivity of this approach achieved by utilizing a combination of ZZ probes in a cocktail probe mixture (40-85 ZZ pairs with approximately 2 to 4.2 kilobase (kb) of genome coverage) and an amplification cascade via sequential hybridization similar to the branched DNA (bDNA) allows for the visualization and discrimination of individual virions or vDNA within the nuclei of infected cells.

There is a growing appreciation of the importance of the FDCn pool as a reservoir from which infection can be reignited on discontinuation of cART. Earlier studies identified the FDCs as cells that HIV [3] and SIV [37] do not infect, but instead binds these viruses via complement receptors on their prolific processes in a state where infectivity is maintained [21, 38]. There is thus a large reservoir [5, 39] of virus well positioned to maintain and reignite infection. Moreover, in the chronic stage of infection, the BCF is a preferential site for continued infection and immunologically privileged in the sense that generally virus-specific CD8+ T cells are not effectively recruited into BCFs [40–43].

For all these reasons, the rapid, sensitive, and quantitative assessment of virus in the BCFs on the FDCn by RNAscope represents a significant methodological advancement to evaluate immunologic therapies and otherwise target this reservoir. When applied in longitudinal analyses in SIV-infected macaques before and during cART, these novel next-generation ISH approaches documented that: 1) the massive viral particle repository trapped on the FDCn within the BCF is significantly diminished with cART, as previously shown in humans [5, 6, 10] but persists at low yet demonstrable levels in most follicles; 2) cART dramatically reduces the number of vRNA+ cells within the TCZ and BCFs, but persistently low levels of vRNA+ cells remain detectable in all compartments within secondary lymphoid tissues; and 3) within the decreased total viral nucleic acid positive cells remaining in the setting of cART, the proportion of vRNA+ and vDNA+ cells decreased in the TCZ, but increased in the BCFs. Collectively, these findings underscore the importance of the BCF as a site of persistent infection and reservoir within the LT compartment, one that must be targeted effectively by immune clearance and other strategies to eradicate infection following transmission or for a functional cure.

Combining RNAscope and DNAscope with immunofluorescent protein detection represents a new enabling technology to characterize the cellular sources of virus production and cellular sanctuaries of persistent infection. We confirm the predominance of infection in T cells during acute SIV infection, and expect that by using antibodies to phenotype the activation state we will find in the future the predominance of infection in ostensibly resting cells in transmission and acute systemic stages, as well as after cART [44, 45]. While we documented visually ingested infected T cells by macrophages, these combined technologies not only distinguished phagocytosis from authentic infection but also enabled assessing potential macrophage reservoirs [46] within LT and other sanctuaries, such as the CNS. Collectively the *in situ* approaches we describe should provide anatomic and cellular information to complement qPCR/qRT-PCR approaches to thereby better understand the establishment and maintenance of viral reservoirs and to evaluate the *in vivo* impact of HIV-1 cure strategies.

## MATERIALS AND METHODS

### Animals and tissue collection

All rhesus macaques (*Macaca mulatta*) were housed and cared for in accordance with the Association for the Assessment and Accreditation of Laboratory Animal Care (AAALAC) standards in AAALAC-accredited facilities. Tissues were utilized from 16 rhesus macaques of Indian origin treated according to protocols approved by the Institutional Animal Care and Use Committee (IACUC) of the National Cancer Institute. Our cohort consisted of the following animals: SIV-negative (n = 2), acute SIV+ (2 wpi; n = 4), chronic SIV+ (8-14 wpi; n = 2), and a longitudinal cohort with samples collected before cART (4 wpi) and after 26 weeks of cART begun at 4 wpi (30 wpi; n = 8). Prior to study inclusion, all animals were free of cercopithicine herpesvirus 1, SIV, simian type-D retrovirus, and simian T-lymphotropic virus type 1. Animals were either intravenously or intrarectally inoculated with SIVmac239 (1 ng p27; 4/6/07 stock; provided by Ronald Desrosiers) or SIVmac239X [47, 48]. As described previously, the cART regimen employed included a daily subcutaneous injection of the co-formulated reverse transcriptase inhibitors (*R*)-9-(2-phosphonylmethoxyypropyl) adenine (PMPA; tenofovir; 20 mg/kg; Gilead Biosciences) and beta-2′, 3′ dideoxy-3′-thia-5-fluorocytidine (FTC; emtricitabine; 40 mg/kg; Gilead Biosciences), and the integrase inhibitors L-870812 (‘812; 20 mg/kg; Merck) and L-900564 (‘564; 10 mg/kg; Merck), each given via the oral route twice daily, and the protease inhibitors darunavir (DRV; 600 mg; Janssen Therapeutics, purchased from the National Institutes of Health [NIH] pharmacy) and ritonavir (RTV; 100 mg; AbbVie, purchased from the NIH pharmacy), each also given via the oral route twice daily as previously described [48]. Lymph nodes were collected at necropsy and/or surgical extraction before (4 wpi) and 26 weeks after cART (30 wpi) and immediately fixed in freshly prepared neutral buffered 4% paraformaldehyde (PFA) for 24 hours at room temperature. After fixation for 24 hours, fixative was replaced with 80% ethanol and tissues were paraffin embedded as previously described [48, 49].

### Plasma viral load, cell-associated viral RNA, and cell-associated viral DNA determination

Plasma viral loads, cell-associated viral RNA, and cell-associated viral DNA determination were performed as previously described [47]. SIV DNA and RNA values were normalized to cell equivalents (CE), determined by co-amplification of a target sequence in the rhesus (or human) CCR5 gene.

### Cell lines and SIV binding experiment

To calibrate our validation studies for detection of vDNA by DNAScope we used well-characterized cell lines containing a reported single integrated provirus, 3D8 T cells (for SIV) and ACH-2 cells (for HIV-1) [22–25]. In proportional reconstitution experiments, 3D8 or ACH-2 cells were mixed with uninfected CEM cells at different ratios (1:0, 4:1, 1:1, 1:4, 0:1) to obtain serial dilutions of cells containing viral genomes in a cell pellet of 10-20 x 10^6^ total cells. Cells were washed in PBS and aliquoted for qPCR or, after fixation in 4% paraformaldehyde (PFA) at room temperature for 24 hours with mild aggregation on a slow speed rocker, for DNAScope analysis. Fixed cells were washed to remove PFA, which was replaced with 80% ethanol followed by gentle mixing. Cells were then washed to remove ethanol and suspended in 4 drops of liquefied Histogel biopsy gel (Fisher Scientific Catalog No.: NC9150318) pre-warmed to 50°C. After mixing in the Histogel, cells were centrifuged at 2,000 x *g* for 5 minutes (using a Fisher Scientific accuSpin™ Micro 17 microcentrifuge) and placed at 4°C until the Histogel solidified, at which point the gel was covered by ethanol 80% and the cell pellet was paraffin embedded.

SupT1 CCR5^HI^ cell binding studies were performed by incubating 10-fold serial dilutions of well-characterized SIV CP-mac virus (stock = 1.12 x 10^-5^ μg SIV capsid) with SupT1 CCR5^HI^ cells (5-10 x 10^6^) for 2 hours on ice to allow virion binding but not internalization, followed by washing 3 times with cold PBS. Cells at each virus dilution were then aliquoted for qRT-PCR or fixation in 4% PFA at room temperature for 24 hours with mild aggregation on a slow speed rocker and processed for Histogel and paraffin embedding for RNAScope analysis, as described above.

### HIV-1 and SIV RNA in situ hybridization

Radiolabeled and chromogenic HIV-1 and SIV *in situ* hybridization (R-ISH and C-ISH, respectively) were performed as previously described with some modifications [15, 17]. In brief for R-ISH, after acetylation with acetic anhydride and dehydration, slides were hybridized at 45°C overnight with a ^35^S-labeled riboprobe containing 0.5 mM aurintricarboxylic acid in the hybridization mix. After extensive washes and ribonuclease treatment, tissue sections were dehydrated, coated in Ilford K5 or Kodak NTB emulsion diluted with glycerol and ammonium acetate, exposed at 4°C for 11 days, and developed and fixed per manufacturer's instructions. Slides were counterstained with hematoxylin, dehydrated, and mounted with Permount.

Chromogenic HIV-1 and SIV *in situ* hybridization (C-ISH) was performed as previously described [17]. In addition, we utilized RNAscope for SIV and HIV-1 detection [50]. Each series of specific SIV and HIV-1 target probes was designed to hybridize to viral RNA in gag, pol, vif, vpr, tat, rev, env, nef, and vpx genes (for SIV) (Supplemental Table 2). In addition to probes for SIV and HIV-1 clade B described in this report, we have generated and validated RNAscope probes for HIV-1 clades A, CRF_AE, C, and D with similar results to SIV (Supplemental Table 2; data not shown). The target probe design strategy was described previously [19]. Briefly, each target probe contained an approximately 25-base region complementary to the corresponding SIV (or HIV-1) plus-RNA strand (transcribed transcripts or whole transcribed genome) of each gene, a spacer sequence, and a 14-base tail sequence (conceptualized as a Z). A pair of target double Z probes (ZZ), each possessing a different type of tail sequence, hybridized contiguously to a target region (approximately 50 bases). The two-tail sequences together formed a 28-base hybridization site that binds to a signal preamplifier, which initiates a signal amplification cascade via sequential hybridization, similar to the branched DNA (bDNA) method described previously [20], followed by chromogenic enzymatic detection (horseradish peroxidase using 3, 3-diaminobenzidine [DAB] or tyramide-cyanine 3.5 [PerkinElmer] or alkaline phosphatase using Fast Red substrate). This approach targeted about 4.5 kb of the viral genome. The double-Z probe design strategy ensures superior background control because it is highly unlikely that a nonspecific hybridization event will juxtapose a pair of target probes along an off-target molecule to form the 28-base hybridization site required for binding of the preamplifier and also because a single 14-base tail sequence will not bind the preamplifier with sufficient strength to result in successful signal amplification. Sections of tissues or cell pellets (4-6 μm) were mounted on Superfrost Plus microscope slides (Fisher Scientific), heated at 60°C for 1 hour, dewaxed in xylenes for 10 minutes, and then placed in ethanol 100% for 5 minutes before air drying. RNAscope was performed as previously described [19]. First, slides were incubated with RNAscope Pretreat 1 reagent (endogenous peroxidase block; ACD) for 10 minutes at room temperature. Heat-induced epitope retrieval was performed by boiling sections in RNAscope Pretreat 2 buffer (a citrate buffer [10 nmol/L, pH 6]; ACD) for 30 minutes, immediately washed in double distilled water, and then dehydrated in 100% ethanol for 5 minutes before air drying. Hydrophobic barrier pen was applied to encircle the section, then the slides were incubated with diluted (1:5) RNAscope pretreat 3 reagent (protease digestion solution; 2.5 ug/mL) for 20 to 25 minutes at 40°C using a HybEZ hybridization oven (ACD). Sections were rinsed 3 times in double distilled water and then incubated with pre-warmed target probes (20 nmol/L of each oligo probe) in hybridization buffer A (6X SSC [1XSSC is 0.15 mol/L NaCl, 0.015 mol/L Na-citrate], 25% formamide, 0.2% lithium dodecyl sulfate, blocking reagents) and incubated for 2 hours at 40°C. Slides were washed in wash buffer (0.1X or 0.05X SSC, 0.03% lithium dodecyl sulfate) and incubated with amplification reagents as described in the RNAscope 2.0 HD detection protocol. Amplifier 1 (2 nmol/L) in hybridization buffer B (20% formamide, 5X SSC, 0.3% lithiumdodecyl sulfate, 10% dextran sulfate, blocking reagents) at 40°C for 30 minutes; Amplifier 2 (a proprietary enhancer to boost detection efficiency) at 40°C for 15 minutes; Amplifier 3 (2 nmol/L) in hybridization buffer B at 40°C for 30 minutes; Amplifier 4 (2 nmol/L) in hybridization buffer C (2X SSC, blocking reagents) at 40°C for 15 minutes; Amplifier 5 (a proprietary signal amplifier) at room temperature for 30 minutes; Amplifier 6 (a proprietary secondary signal amplifier) at room temperature for 15 minutes. After each hybridization step, slides were washed with wash buffer three times at room temperature. Before detection, the slides were rinsed one time in 1X TBS Tween-20 (0.05% v/v). Amplification 6 contained alkaline phosphatase (or horseradish peroxidase) labels, and chromogenic detection was performed using FastRed as substrate to generate red signal, DAB to generate a brown signal, or tyramide-cyanine 3.5 (PerkinElmer) for fluorescence detection. Red chromogen development was performed following the RNAscope 2.0 HD detection protocol and reagents, brown chromogen development using ImmPACT™ DAB (Vector Laboratories), and fluorescent detection using tyramide-cyanine 3.5 Plus (PerkinElmer) (chromogen incubation time varied between 2 and 8 minutes). Slides were counterstained with haematoxylin or DAPI (4′,6-diamidino-2-phenylin-dole) and mounted in Permount (Fisher Scientific) or Prolong^®^ Gold (ThermoFisher Scientific). Slides mounted in Permount were scanned at high magnification (×400) using the ScanScope AT2 System (Aperio Technologies), yielding high-resolution data from the entire tissue section. Fluorescent slides mounted with Prolong^®^ Gold (Invitrogen) were imaged on an Olympus FV10i confocal microscope using a 60x phase contrast oil-immersion objective (NA 1.35) and applying a sequential mode to separately capture the fluorescence from the different fluorochromes at an image resolution of 1024 x 1024 pixels.

### HIV-1 and SIV DNA in situ hybridization

Viral DNA detection (DNAscope) was performed by utilizing the sense probe targeting the reverse strand for each viral lineage of interest or cellular DNA utilizing a sense probe targeting the complementary CCR5 strand (Supplemental Table 2; data not shown). We modified the RNA-scope procedure by adding an RNase tissue pretreatment step (with ribonucleases A [25 μg/ml; Fisher Scientific] and T_1_ [25 units/ml; Roche Diagnostics] in 1× Tris-buffered saline [TBS; Boston BioProducts] containing 0.05% Tween-20 [TBS-Tw] for 30 minutes at 37°C) following the RNA-scope pretreat three step, which was followed by a short denaturation step in which we incubated the slides at 60°C with warmed (60°C) sense probes for 10 to 15 minutes, and then immediately transferred the hybridization chamber to an oven set at 40°C and hybridized overnight (between 18 to 21 hours). Amplification and detection were performed as described for RNAscope previously, using 0.5X wash buffer for all washing steps. DNAscope validation was performed on 3D8 and ACH-2 cells diluted in uninfected CEM cells (generated as described previously) to make cell preparations at different concentrations ranging from 0% 3D8 or ACH2 cells to 100% 3D8 or ACH2 cells. Cell pellets were embedded in paraffin blocks, after which 4 to 6 μm sections were cut, and SIV DNA *in situ* hybridization was performed using DNAscope.

### Duplex vRNA and vDNA in situ hybridization

Simultaneous visualization of both vRNA and vDNA was performed by combining two custom probe sets—one sense (targeting the vDNA coding strand) and one anti-sense (targeting vRNA transcripts)—covering different regions of the viral genome (Supplemental Table 3) using the DNAscope protocol (without RNase treatment). We utilized a customized RNAscope 2.0 HD Multiplex detection protocol (ACD) for detecting vRNA (Fast Red) followed by detection of vDNA (DAB-Brown) allowing for the visualization of both vRNA and vDNA in the same tissue section.

### Simultaneous viral DNA and immunofluorescent detection for confocal phenotypic analysis

To immunophenotype the cells containing vDNA, we combined DNAscope detection by red chromogen or Tyramide Signal Amplification (TSA™) Plus Cy3.5 with immunofluorescence targeting cell markers using rabbit monoclonal anti-CD4 (1:200; clone EPR6855; Abcam) or rabbit monoclonal anti-CD3 (1:100; clone SP7; Labvision/Thermo Scientific) for T-lineage cells and a cocktail of 2 antibodies: CD68 (1:500; clone KP1; Biocare Medical, Inc.) and CD163 (1:500; clone 10D6; Novocastra/Leica Microsystems Inc.) for myeloid-lineage cells. We performed DNAscope following the protocol described above but after the last amplification step, vDNA was not immediately developed with red chromogen or TSA Plus Cy3.5. Instead slides were directly incubated over night at 4°C with antibodies to phenotype T- and myeloid-lineage cells. Slides were washed, incubated with secondary donkey anti-mouse/rabbit IgG-Alexa 488 or Alexa 647 (all from Molecular Probes/ThermoFisher Scientific) for 1 hour at room temperature, and washed 2 times for 5 minutes in TBS + tween (0.05% v/v). To decrease autofluorescence, the tissues were incubated with Sudan Black solution (0.1% in 80% ethanol [ENG Scientific, Inc.] + 1x TBS); for 20 to 30 minutes at room temperature, washed, counterstained with DAPI (RTU; ACD) for 10 minutes, washed, and then developed with either Fast Red chromogen or TSA Plus Cy3.5 to reveal vDNA (development varied depending on the tissue type, but was typically 8 minutes), washed in TBS and cover slipped with #1.5 GOLD SEAL^®^ cover glass (EMS) using Prolong^®^ Gold reagent (Invitrogen).

### Quantitative image analysis

To quantify vRNA copies from R-ISH stained tissues, photographic images using epifluorescence were taken with a digital camera, and the TIFF images were used to analyze the area occupied by silver grains using Photoshop (Adobe Systems, San Jose, California) with Fovea Pro 4 (Reindeer Graphics, Asheville, North Carolina), corrected by the background silver grain density of the slide. Section weights were estimated from their volume (5-μm thickness × their area). The number of copies of vRNA was calculated as follows: silver grains observed/cell × 2 disintegrations/grain × 1 Ci ÷ 2.2x10^12^ disintegrations/min ÷ exposure time (min) ÷ specific activity of probe (Ci/mmole probe RNA) × 6.02 × 10^20^ copies/mmole **=** vRNA copies/cell) [5, 6, 10]. For virions associated with the follicular dendritic cell network (FDCn), silver grains were enumerated over follicles, excluding grains over vRNA+ cells, and the number of vRNA copies in the follicle estimated as just described, dividing by 2 based on 2 vRNA copies/virion to estimate the number of virions.

To quantify RNAscope stained tissues, whole slides were scanned at 40× magnification with an AT2 slide scanner (Aperio Technologies, Vista, California), and regions of interest (ROIs), including B cell follicles (BCF) were saved as TIFF images for analysis. ROIs were analyzed with Photo-shop CS6 (Adobe Systems, San Jose, California) using Noiseware 5 (Imagenomic, Vienna, Virginia) for noise reduction and Fovea Pro 4 (Reindeer Graphics, Asheville, North Carolina) for the analysis. To estimate the number of virions in BCF, ROIs were segmented by reducing noise with Noiseware, duplicating the ROI image, converting the duplicate from RGB to CMYK color mode, thresholding the magenta channel, and then copying and pasting the revised image as a new layer on the original image; the threshold value was determined empirically for each set of slides. To refine the segmentation, Fovea's fill holes command was used to consolidated positive areas, and then the reject features command was applied to remove any objects < 2 pixels or touching an edge of the image. Stained areas of each ROI were recorded with Fovea's area fraction command. Image quantitation data were recorded in text files, and analyzed images were saved in Photoshop format. The fraction of BCF ROIs that stained for vRNA by RNAscope was divided by the average virion size calculated from discretely resolved virions from both SupT1 CCR5+ cells and LTs stained with RNAscope and Fast Red (average virion size = 0.9695 μm^2^).

To quantify the number of productively infected vRNA+ cells from RNAscope stained tissues, Fast Red staining in an ROI was segmented by reducing noise with Noiseware, duplicating the ROI image, converting the duplicate from RGB to CMYK color mode, thresholding the magenta channel, and then copying and pasting the revised image as a new layer on the original image. The segmentation was refined by morphological opening and closing using Fovea's Euclidean distance map-based morphology operations; when counting positive cells, a morphological opening removed small objects, and then a closing consolidated positive areas within cells. Watershed segmentation was also performed to segment individual stained objects and the Fast Red-positive objects were counted with Fovea. The data were recorded in a text file, and the analyzed image was saved in Photoshop format. The number of vRNA+ cells was calculated based on area (mm^2^) or per 10^5^ cells. Nuclei in an ROI were segmented by thresholding the red channel with the Pun algorithm in Fovea, morphological opening, watershed segmentation, and then counting the segmented objects. The number of vRNA+ cells was divided by the estimated number of cells determined by the nuclei segmentation in that image and multiplied by 10^5^.

The number of vDNA+ cells was determined by either manually counting by at least two individuals who were blinded to the proportion of 3D8 or ACH2 cells on each slide or quantified using a similar approach to virion quantification with noise reduction and thresholding the CMYK magenta channel as above, and rejecting objects < 9 pixels or touching an edge of the image.

### Statistical analyses

Statistical analyses were performed using Prism (v6.0; GraphPad Software, La Jolla, California). The Mann–Whitney U test was used for comparisons between groups; the Wilcoxon matched-pairs Signed-Rank test was used for comparisons within groups, and the Pearson's correlation coefficient (Pearson's r) was used to measure of the linear correlation between two variables after log10 transformation of the data. Averaged data were presented as the arithmetic mean ± standard error of the mean (SEM). *P* values less than 0.05 were considered significant.

## SUPPLEMENTARY MATERIALS

**Supplemental Table 1.** Rate of false-positive detection for vRNA+ cells and virions by RNAscope

Chronic SIV+ RMs	Total cell counts	Total area (mm^2^)	Number of HPFs (200-400x)	vRNA+ cells	vRNA+ / 10^5^ cells	Total virions	Virions / 10^5^ cells
*DCEA*	108,134	7.244	29	629	582.138	523	484
*DCEW*	37,583	2.748	11	443	1,177.712	73,278	194,977
F55	80,563	1.750	7	67	83.165	210,629	261,446
**Total**	**226,280**	**11.742**	**47**	**1,139**	**1,843**	**284,430**	**456,907**

SIV-negative RMs	Total cell counts	Total area (mm^2^)	Number of HPFs (200-400x)	vRNA+ cells	False positive rate (vRNA+/10^5^ cells)	Total virions	False positive rate (Virions/10^5^ cells)
*DCEA*	103,198	6.744	27	1	0.969	2	2
*DCEW*	127,202	8.992	36	1	0.786	1	1
DCLJ	143,840	9.242	37	0	0.000	1	1
DCT2	115,141	7.244	29	0	0.000	0	0
P375	219,297	13.478	54	0	0.000	0	0
P375	582,006	13.500	54	0	0.000	0	0
P380	783,341	15.999	64	0	0.000	2	0
**Total**	**1,970,827**	**68.455**	**274**	**2**	**0.101**	**4**	**0.203**

**Supplemental Table 2.** Single-plex RNAscope and DNAscope probes

Single-Plex RNA/DNAscope Probe Sets			
Name	ACD catalog #	Number of ZZ	Description
SIVmac239-C1 (Anti-sense)	312811-C1	83	Anti-sense probe targeting within 1251-9420bp of D01065.1 (gag, pol, vif, vpx, vpr, tat, env, and nef)
SIVmac239-SENSE-C1	314071-C1	83	Sense probe targeting reverse strand within 1251-9420bp of D01065.1 (gag, pol, vif, vpx, vpr, tat, env, and nef)
RM-CCR5-SENSE-C1	416151-C1	14	Sense probe targeting the complement strand within 36-1271bp of NM_001042773.2
V-HIV1-Clade A-C1 (Anti-sense)	416101-C1	80	Anti-sense probe targeting within 879-7629bp of HIV-1 Clade A Consen-sus (gag, pol, vif, vpr, tat, rev, vpu, env, and nef)
V-HIV1-Clade A-SENSE	426341-C1	80	Sense probe targeting reverse strand within 879-7629bp of HIV-1 Clade A consensus (gag, pol, vif, vpr, tat, rev, vpu, env, and nef)
V-HIV1-Clade B-C1 (Anti-sense)	416111-C1	78	Anti-sense probe targeting within 854-8291bp of AF324493.2, HIV-1 Clade B NL4-3 (gag, pol, vif, vpr, tat, rev, vpu, env, and nef)
V-HIV1-Clade B-SENSE	425531-C1	78	Sense probe targeting reverse strand within 854-8291bp of AF324493.2, HIV-1 Clade B NL4-3 (gag, pol, vif, vpr, tat, rev, vpu, env, and nef)
V-HIV1-Clade D-C1 (Anti-sense)	416121-C1	76	Anti-sense probe targeting within 894-7697bp of HIV-1 Clade D Consen-sus (gag, pol, vif, vpr, tat, rev, vpu, env, nef)
V-HIV1-Clade D-SENSE	426351-C1	76	Sense probe targeting reverse strand within 894-7697bp of HIV-1 Clade D Consensus (gag, pol, vif, vpr, tat, rev, vpu, env, nef)

**Supplemental Table 3.** Duplex RNAscope and DNAscope probes

Multi-Plex RNA/DNAscope Probe Sets			
Name	ACD catalog #	Number of ZZ	Description
V-SIVmac239-gag-pol-Sense-C1	416141-C1	40	Sense probe targeting reverse strand within 1251-4093bp of D01065.1 (gag and pol)
V-SIVmac239-vif-env-nef-tar-C2 (Anti-sense)	416131-C2	47	Anti-sense probe targeting within 5381-10257bp of D01065.1 (vif, vpx, vpr, tat, env, nef, and the TAR element)
V-HIV1-Clade_B-gag-pol-sense-C1	444051-C1	40	Sense probe targeting reverse strand within 854-3940bp of AF324493.2, HIV-1 Clade B NL4-3 (gag and pol)
V-HIV1-Clade_B-vif-vpr-tat-rev-vpu-env-nef-tar-C2 (Anti-sense)	444061-C2	40	Anti-sense probe targeting within 5042-9673bp of AF324493.2, HIV-1 Clade B NL4-3 (vif, vpr, tat, env, nef, and the TAR element)
V-HIV1-Clade_C-gag-pol-sense-C1	444021-C1	48	Sense probe targeting reverse strand within 888- 5032bp of HIV-1 Clade C consensus sequence (gag and pol)
V-HIV1-Clade_C-vif-vpr-rev-vpu-env-nef-tar-C2 (Anti-sense)	444041-C2	49	Anti-sense probe targeting within 5078-9698bp of HIV-1 Clade C consensus sequence (vif, vpr, tat, env, nef, and the TAR element)
V-HIV1-Clade_AE-gag-pol-sense-C1	444011-C1	55	Sense probe targeting reverse strand within 890-4812bp of AF259954.1, HIV-1 Clade AE (gag and pol)
V-HIV1-Clade_AE-vif-vpr-tat-rev-vpu-env-nef-tar-C2 (Anti-sense)	444031-C2	57	Anti-sense probe targeting within 5052-9694bp of AF259954.1, HIV-1 Clade AE (vif, vpr, tat, env, nef, and the T

## References

[1] Archin NM, Sung JM, Garrido C, Soriano-Sarabia N, Margolis DM Eradicating HIV-1 infection: seeking to clear a persistent pathogen. Nat Rev Microbiol. 2014;12(11):750–64. PubMed PMID: 25402363. Pubmed Central PMCID: 4383747. doi: 10.1038/nrmicro3352 25402363 PMC4383747

[2] Pantaleo G, Graziosi C, Butini L, Pizzo PA, Schnittman SM, Kotler DP, Fauci AS Lymphoid organs function as major reservoirs for human immunodeficiency virus. Proc Natl Acad Sci U S A. 1991;88(21):9838–42. PubMed PMID: 1682922. Pubmed Central PMCID: PMC52816. Epub 1991/11/01. 1682922 10.1073/pnas.88.21.9838 PMC52816

[3] Embretson J, Zupancic M, Ribas JL, Burke A, Racz P, Tenner-Racz K, Haase AT Massive covert infection of helper T lymphocytes and macrophages by HIV during the incubation period of AIDS. Nature. 1993;362(6418):359–62. PubMed PMID: 8096068. doi: 10.1038/362359a0 8096068

[4] Chun TW, Finzi D, Margolick J, Chadwick K, Schwartz D, Siliciano RF In vivo fate of HIV-1-infected T cells: quantitative analysis of the transition to stable latency. Nat Med. 1995;1(12):1284–90. PubMed PMID: 7489410. Epub 1995/12/01. 7489410 10.1038/nm1295-1284

[5] Haase AT, Henry K, Zupancic M, Sedgewick G, Faust RA, Melroe H, Cavert W, Gebhard K, Staskus K, Zhang ZQ, Dailey PJ, Balfour HH Jr., Erice A, Perelson AS Quantitative image analysis of HIV-1 infection in lymphoid tissue. Science. 1996;274(5289):985–9. PubMed PMID: 8875941. 8875941 10.1126/science.274.5289.985

[6] Cavert W, Notermans DW, Staskus K, Wietgrefe SW, Zupancic M, Gebhard K, Henry K, Zhang ZQ, Mills R, McDade H, Schuwirth CM, Goudsmit J, Danner SA, Haase AT Kinetics of response in lymphoid tissues to antiretroviral therapy of HIV-1 infection. Science. 1997;276(5314):960–4. PubMed PMID: 9139661. Epub 1997/05/09. 9139661 10.1126/science.276.5314.960

[7] Finzi D, Hermankova M, Pierson T, Carruth LM, Buck C, Chaisson RE, Quinn TC, Chadwick K, Margolick J, Brookmeyer R, Gallant J, Markowitz M, Ho DD, Richman DD, Siliciano RF Identification of a reservoir for HIV-1 in patients on highly active antiretroviral therapy. Science. 1997;278(5341):1295–300. PubMed PMID: 9360927. Epub 1997/11/21. 9360927 10.1126/science.278.5341.1295

[8] Reinhart TA, Rogan MJ, Huddleston D, Rausch DM, Eiden LE, Haase AT Simian immunodeficiency virus burden in tissues and cellular compartments during clinical latency and AIDS. J Infect Dis. 1997;176(5):1198–208. PubMed PMID: 9359719. 9359719 10.1086/514113

[9] Haase AT Population biology of HIV-1 infection: viral and CD4+ T cell demographics and dynamics in lymphatic tissues. Annu Rev Immunol. 1999;17:625–56. PubMed PMID: 10358770. doi: 10.1146/annurev.immunol.17.1.625 10358770

[10] Schacker T, Little S, Connick E, Gebhard-Mitchell K, Zhang ZQ, Krieger J, Pryor J, Havlir D, Wong JK, Richman D, Corey L, Haase AT Rapid accumulation of human immunodeficiency virus (HIV) in lymphatic tissue reservoirs during acute and early HIV infection: implications for timing of antiretroviral therapy. J Infect Dis. 2000;181(1):354–7. PubMed PMID: 10608788. doi: 10.1086/315178 10608788

[11] Schacker T, Little S, Connick E, Gebhard K, Zhang ZQ, Krieger J, Pryor J, Havlir D, Wong JK, Schooley RT, Richman D, Corey L, Haase AT Productive infection of T cells in lymphoid tissues during primary and early human immunodeficiency virus infection. J Infect Dis. 2001;183(4):555–62. PubMed PMID: 11170980. doi: 10.1086/318524 11170980

[12] Han Y, Wind-Rotolo M, Yang HC, Siliciano JD, Siliciano RF Experimental approaches to the study of HIV-1 latency. Nat Rev Microbiol. 2007;5(2):95–106. PubMed PMID: 17224919. doi: 10.1038/nrmicro1580 17224919

[13] Rouzioux C, Richman D How to best measure HIV reservoirs? Curr Opin HIV AIDS. 2013;8(3):170–5. PubMed PMID: 23564004. Pubmed Central PMCID: PMC3763804. Epub 2013/04/09. doi: 10.1097/COH.0b013e32835fc619 23564004 PMC3763804

[14] Nicol A, Nuovo GJ Detection of HIV-1 provirus and RNA by in situ amplification. Methods Mol Biol. 2005;304:171–82. PubMed PMID: 16061974. doi: 10.1385/1-59259-907-9:171 16061974

[15] Fletcher CV, Staskus K, Wietgrefe SW, Rothenberger M, Reilly C, Chipman JG, Beilman GJ, Khoruts A, Thorkelson A, Schmidt TE, Anderson J, Perkey K, Stevenson M, Perelson AS, Douek DC, Haase AT, Schacker TW Persistent HIV-1 replication is associated with lower antiretroviral drug concentrations in lymphatic tissues. Proc Natl Acad Sci U S A. 2014;111(6):2307–12. PubMed PMID: 24469825. Pubmed Central PMCID: PMC3926074. Epub 2014/01/29. eng. doi: 10.1073/pnas.1318249111 24469825 PMC3926074

[16] Rothenberger MK, Keele BF, Wietgrefe SW, Fletcher CV, Beilman GJ, Chipman JG, Khoruts A, Estes JD, Anderson J, Callisto SP, Schmidt TE, Thorkelson A, Reilly C, Perkey K, Reimann TG, Utay NS, Nganou Makamdop K, Stevenson M, Douek DC, Haase AT, Schacker TW Large number of rebounding/founder HIV variants emerge from multifocal infection in lymphatic tissues after treatment interruption. Proc Natl Acad Sci U S A. 2015;112(10):E1126–34. PubMed PMID: 25713386. Pubmed Central PMCID: PMC4364237. Epub 2015/02/26. doi: 10.1073/pnas.1414926112 25713386 PMC4364237

[17] Brenchley JM, Vinton C, Tabb B, Hao XP, Connick E, Paiardini M, Lifson JD, Silvestri G, Estes JD Differential infection patterns of CD4+ T cells and lymphoid tissue viral burden distinguish progressive and nonprogressive lentiviral infections. Blood. 2012;120(20):4172–81. PubMed PMID: 22990012. Pubmed Central PMCID: 3501715. doi: 10.1182/blood-2012-06-437608 22990012 PMC3501715

[18] Micci L, Alvarez X, Iriele RI, Ortiz AM, Ryan ES, McGary CS, Deleage C, McAtee BB, He T, Apetrei C, Easley K, Pahwa S, Collman RG, Derdeyn CA, Davenport MP, Estes JD, Silvestri G, Lackner AA, Paiardini M CD4 depletion in SIV-infected macaques results in macrophage and microglia infection with rapid turnover of infected cells. PLoS Pathog. 2014;10(10):e1004467 PubMed PMID: 25356757. Pubmed Central PMCID: PMC4214815. doi: 10.1371/journal.ppat.1004467 25356757 PMC4214815

[19] Wang F, Flanagan J, Su N, Wang LC, Bui S, Nielson A, Wu X, Vo HT, Ma XJ, Luo Y RNAscope: a novel in situ RNA analysis platform for formalin-fixed, paraffin-embedded tissues. J Mol Diagn. 2012;14(1):22–9. PubMed PMID: 22166544. Pubmed Central PMCID: PMC3338343. doi: 10.1016/j.jmoldx.2011.08.002 22166544 PMC3338343

[20] Player AN, Shen LP, Kenny D, Antao VP, Kolberg JA Single-copy gene detection using branched DNA (bDNA) in situ hybridization. J Histochem Cytochem. 2001;49(5):603–12. PubMed PMID: 11304798. Epub 2001/04/17. 11304798 10.1177/002215540104900507

[21] Heesters BA, Lindqvist M, Vagefi PA, Scully EP, Schildberg FA, Altfeld M, Walker BD, Kaufmann DE, Carroll MC Follicular Dendritic Cells Retain Infectious HIV in Cycling Endosomes. PLoS Pathog. 2015;11(12):e1005285 PubMed PMID: 26623655. Pubmed Central PMCID: PMC4666623. doi: 10.1371/journal.ppat.1005285 26623655 PMC4666623

[22] Nishimura Y, Sadjadpour R, Mattapallil JJ, Igarashi T, Lee W, Buckler-White A, Roederer M, Chun TW, Martin MA High frequencies of resting CD4+ T cells containing integrated viral DNA are found in rhesus macaques during acute lentivirus infections. Proc Natl Acad Sci U S A. 2009;106(19):8015–20. PubMed PMID: 19416840. Pubmed Central PMCID: PMC2683103. doi: 10.1073/pnas.0903022106 19416840 PMC2683103

[23] Bertram S, Hufert FT, Neumann-Haefelin D, von Laer D Detection of DNA in single cells using an automated cell deposition unit and PCR. Biotechniques. 1995;19(4):616–20. PubMed PMID: 8777056. Epub 1995/10/01. 8777056

[24] Stanfield-Oakley SA, Griffith JD Nucleosomal arrangement of HIV-1 DNA: maps generated from an integrated genome and an EBV-based episomal model. J Mol Biol. 1996;256(3):503–16. PubMed PMID: 8604134. doi: 10.1006/jmbi.1996.0104 8604134

[25] Clouse KA, Powell D, Washington I, Poli G, Strebel K, Farrar W, Barstad P, Kovacs J, Fauci AS, Folks TM Monokine regulation of human immunodeficiency virus-1 expression in a chronically infected human T cell clone. J Immunol. 1989;142(2):431–8. PubMed PMID: 2463307. 2463307

[26] O'Doherty U, Swiggard WJ, Jeyakumar D, McGain D, Malim MH A sensitive, quantitative assay for human immunodeficiency virus type 1 integration. J Virol. 2002;76(21):10942–50. PubMed PMID: 12368337. Pubmed Central PMCID: PMC136638. 12368337 10.1128/JVI.76.21.10942-10950.2002 PMC136638

[27] Kim H, Terazono H, Takei H, Yasuda K Cup-shaped superparamagnetic hemispheres for size-selective cell filtration. Sci Rep. 2014;4:6362 PubMed PMID: 25219418. Pubmed Central PMCID: 4163672. doi: 10.1038/srep06362 25219418 PMC4163672

[28] Del Portillo A, Tripodi J, Najfeld V, Wodarz D, Levy DN, Chen BK Multiploid inheritance of HIV-1 during cell-to-cell infection. J Virol. 2011;85(14):7169–76. PubMed PMID: 21543479. Pubmed Central PMCID: PMC3126592. doi: 10.1128/JVI.00231-11 21543479 PMC3126592

[29] Russell RA, Martin N, Mitar I, Jones E, Sattentau QJ Multiple proviral integration events after virological synapse-mediated HIV-1 spread. Virology. 2013;443(1):143–9. PubMed PMID: 23722103. doi: 10.1016/j.virol.2013.05.005 23722103

[30] Calantone N, Wu F, Klase Z, Deleage C, Perkins M, Matsuda K, Thompson EA, Ortiz AM, Vinton CL, Ourmanov I, Lore K, Douek DC, Estes JD, Hirsch VM, Brenchley JM Tissue myeloid cells in SIV-infected primates acquire viral DNA through phagocytosis of infected T cells. Immunity. 2014;41(3):493–502. PubMed PMID: 25238099. Pubmed Central PMCID: PMC4241569. doi: 10.1016/j.immuni.2014.08.014 25238099 PMC4241569

[31] Xing S, Siliciano RF Targeting HIV latency: pharmacologic strategies toward eradication. Drug Discov Today. 2013;18(11-12):541–51. PubMed PMID: 23270785. Pubmed Central PMCID: PMC3672351. doi: 10.1016/j.drudis.2012.12.008 23270785 PMC3672351

[32] Shan L, Siliciano RF From reactivation of latent HIV-1 to elimination of the latent reservoir: the presence of multiple barriers to viral eradication. Bioes-says. 2013;35(6):544–52. PubMed PMID: 23613347. Pubmed Central PMCID: PMC4386637. doi: 10.1002/bies.201200170 PMC4386637 23613347

[33] Chun TW, Moir S, Fauci AS HIV reservoirs as obstacles and opportunities for an HIV cure. Nat Immunol. 2015;16(6):584–9. PubMed PMID: 25990814. Epub 2015/05/21. doi: 10.1038/ni.3152 25990814

[34] Tenner-Racz K, Racz P, Gartner S, Ramsauer J, Dietrich M, Gluckman JC, Popovic M Ultrastructural analysis of germinal centers in lymph nodes of patients with HIV-1-induced persistent generalized lymphadenopathy: evidence for persistence of infection. Prog AIDS Pathol. 1989;1:29–40. PubMed PMID: 2491247. 2491247

[35] Lorenzo-Redondo R, Fryer HR, Bedford T, Kim EY, Archer J, Kosakovsky Pond SL, Chung YS, Penugonda S, Chipman JG, Fletcher CV, Schacker TW, Malim MH, Rambaut A, Haase AT, McLean AR, Wolinsky SM Persistent HIV-1 replication maintains the tissue reservoir during therapy. Nature. 2016;530(7588):51–6. PubMed PMID: 26814962. doi: 10.1038/nature16933 26814962 PMC4865637

[36] Haase AT, Retzel EF, Staskus KA Amplification and detection of lentiviral DNA inside cells. Proc Natl Acad Sci U S A. 1990;87(13):4971–5. PubMed PMID: 2164214. Pubmed Central PMCID: PMC54243. 2164214 10.1073/pnas.87.13.4971 PMC54243

[37] Reinhart TA, Rogan MJ, Viglianti GA, Rausch DM, Eiden LE, Haase AT A new approach to investigating the relationship between productive infection and cytopathicity in vivo. Nat Med. 1997;3(2):218–21. PubMed PMID: 9018242. Epub 1997/02/01. 9018242 10.1038/nm0297-218

[38] Smith BA, Gartner S, Liu Y, Perelson AS, Stilianakis NI, Keele BF, Kerkering TM, Ferreira-Gonzalez A, Szakal AK, Tew JG, Burton GF Persistence of infectious HIV on follicular dendritic cells. J Immunol. 2001;166(1):690–6. PubMed PMID: 11123354. 11123354 10.4049/jimmunol.166.1.690

[39] Spiegel H, Herbst H, Niedobitek G, Foss HD, Stein H Follicular dendritic cells are a major reservoir for human immunodeficiency virus type 1 in lymphoid tissues facilitating infection of CD4+ T-helper cells. Am J Pathol. 1992;140(1):15–22. PubMed PMID: 1530997. Pubmed Central PMCID: PMC1886262. 1530997 PMC1886262

[40] Connick E, Folkvord JM, Lind KT, Rakasz EG, Miles B, Wilson NA, Santiago ML, Schmitt K, Stephens EB, Kim HO, Wagstaff R, Li S, Abdelaal HM, Kemp N, Watkins DI, MaWhinney S, Skinner PJ Compartmentalization of simian immunodeficiency virus replication within secondary lymphoid tissues of rhesus macaques is linked to disease stage and inversely related to localization of virus-specific CTL. J Immunol. 2014;193(11):5613–25. PubMed PMID: 25362178. Pubmed Central PMCID: PMC4239212. Epub 2014/11/02. doi: 10.4049/jimmunol.1401161 25362178 PMC4239212

[41] Connick E, Mattila T, Folkvord JM, Schlichtemeier R, Meditz AL, Ray MG, McCarter MD, Mawhinney S, Hage A, White C, Skinner PJ CTL fail to accumulate at sites of HIV-1 replication in lymphoid tissue. J Immunol. 2007;178(11):6975–83. PubMed PMID: 17513747. 17513747 10.4049/jimmunol.178.11.6975

[42] Folkvord JM, Armon C, Connick E Lymphoid follicles are sites of heightened human immunodeficiency virus type 1 (HIV-1) replication and reduced antiretroviral effector mechanisms. AIDS Res Hum Retroviruses. 2005;21(5):363–70. PubMed PMID: 15929698. doi: 10.1089/aid.2005.21.363 15929698

[43] Fukazawa Y, Lum R, Okoye AA, Park H, Matsuda K, Bae JY, Hagen SI, Shoemaker R, Deleage C, Lucero C, Morcock D, Swanson T, Legasse AW, Axthelm MK, Hesselgesser J, Geleziunas R, Hirsch VM, Edlefsen PT, Piatak M Jr., Estes JD, Lifson JD, Picker LJ B cell follicle sanctuary permits persistent productive simian immunodeficiency virus infection in elite controllers. Nat Med. 2015;21(2):132–9. PubMed PMID: 25599132. Pubmed Central PMCID: PMC4320022. doi: 10.1038/nm.3781 25599132 PMC4320022

[44] Zhang ZQ, Wietgrefe SW, Li Q, Shore MD, Duan L, Reilly C, Lifson JD, Haase AT Roles of substrate availability and infection of resting and activated CD4+ T cells in transmission and acute simian immunodeficiency virus infection. Proc Natl Acad Sci U S A. 2004;101(15):5640–5. PubMed PMID: 15064398. Pubmed Central PMCID: PMC397458. doi: 10.1073/pnas.0308425101 15064398 PMC397458

[45] Li Q, Duan L, Estes JD, Ma ZM, Rourke T, Wang Y, Reilly C, Carlis J, Miller CJ, Haase AT Peak SIV replication in resting memory CD4+ T cells depletes gut lamina propria CD4+ T cells. Nature. 2005;434(7037):1148–52. PubMed PMID: 15793562. doi: 10.1038/nature03513 15793562

[46] Abbas W, Tariq M, Iqbal M, Kumar A, Herbein G Eradication of HIV-1 from the macrophage reservoir: an uncertain goal? Viruses. 2015;7(4):1578–98. PubMed PMID: 25835530. Pubmed Central PMCID: PMC4411666. doi: 10.3390/v7041578 25835530 PMC4411666

[47] Del Prete GQ, Park H, Fennessey CM, Reid C, Lipkey L, Newman L, Oswald K, Kahl C, Piatak M Jr., Quinones OA, Alvord WG, Smedley J, Estes JD, Lifson JD, Picker LJ, Keele BF Molecularly tagged simian immunodeficiency virus SIVmac239 synthetic swarm for tracking independent infection events. J Virol. 2014;88(14):8077–90. PubMed PMID: 24807714. Pubmed Central PMCID: PMC4097795. doi: 10.1128/JVI.01026-14 24807714 PMC4097795

[48] Del Prete GQ, Shoemaker R, Oswald K, Lara A, Trubey CM, Fast R, Schneider DK, Kiser R, Coalter V, Wiles A, Wiles R, Freemire B, Keele BF, Estes JD, Quinones OA, Smedley J, Macallister R, Sanchez RI, Wai JS, Tan CM, Alvord WG, Hazuda DJ, Piatak M Jr., Lifson JD Effect of suberoylanilide hydroxamic acid (SAHA) administration on the residual virus pool in a model of combination antiretroviral therapy-mediated suppression in SIVmac239-infected indian rhesus macaques. Antimicrob Agents Chemother. 2014;58(11):6790–806. PubMed PMID: 25182644. Pubmed Central PMCID: PMC4249371. doi: 10.1128/AAC.03746-14 25182644 PMC4249371

[49] Hao XP, Lucero CM, Turkbey B, Bernardo ML, Morcock DR, Deleage C, Trubey CM, Smedley J, Klatt NR, Giavedoni LD, Kristoff J, Xu A, Del Prete GQ, Keele BF, Rao SS, Alvord WG, Choyke PL, Lifson JD, Brenchley JM, Apetrei C, Pandrea I, Estes JD Experimental colitis in SIV-uninfected rhesus macaques recapitulates important features of pathogenic SIV infection. Nat Commun. 2015;6:8020 PubMed PMID: 26282376. Pubmed Central PMCID: PMC4544774. doi: 10.1038/ncomms9020 26282376 PMC4544774

[50] Smedley J, Turkbey B, Bernardo ML, Del Prete GQ, Estes JD, Griffiths GL, Kobayashi H, Choyke PL, Lifson JD, Keele BF Tracking the luminal exposure and lymphatic drainage pathways of intravaginal and intrarectal inocula used in nonhuman primate models of HIV transmission. PLoS One. 2014;9(3):e92830 PubMed PMID: 24667371. Pubmed Central PMCID: PMC3965472. doi: 10.1371/journal.pone.0092830 24667371 PMC3965472

